# Phylogeny of Elatinaceae and the Tropical Gondwanan Origin of the Centroplacaceae(Malpighiaceae, Elatinaceae) Clade

**DOI:** 10.1371/journal.pone.0161881

**Published:** 2016-09-29

**Authors:** Liming Cai, Zhenxiang Xi, Kylee Peterson, Catherine Rushworth, Jeremy Beaulieu, Charles C. Davis

**Affiliations:** 1 Department of Organismic and Evolutionary Biology, Harvard University Herbaria, 22 Divinity Avenue, Cambridge, Massachusetts, 02138, United States of America; 2 Department of Ecology and Evolutionary Biology, University of Tennessee, Knoxville, TN, 37996, United States of America; Institute of Botany, CHINA

## Abstract

The flowering plant family Elatinaceae is a widespread aquatic lineage inhabiting temperate and tropical latitudes, including ∼35(-50) species. Its phylogeny remains largely unknown, compromising our understanding of its systematics. Moreover, this group is particularly in need of attention because the biogeography of most aquatic plant clades has yet to be investigated, resulting in uncertainty about whether aquatic plants show histories that deviate from terrestrial plants. We inferred the phylogeny of Elatinaceae from four DNA regions spanning 59 accessions across the family. An expanded sampling was used for molecular divergence time estimation and ancestral area reconstruction to infer the biogeography of Elatinaceae and their closest terrestrial relatives, Malpighiaceae and Centroplacaceae. The two genera of Elatinaceae, *Bergia* and *Elatine*, are monophyletic, but several traditionally recognized groups within the family are non-monophyletic. Our results suggest two ancient biogeographic events in the Centroplacaceae(Malpighiaceae, Elatinaceae) clade involving western Gondwana, while Elatinaceae shows a more complicated biogeographic history with a high degree of continental endemicity. Our results indicate the need for further taxonomic investigation of Elatinaceae. Further, our study is one of few to implicate ancient Gondwanan biogeography in extant angiosperms, especially significant given the Centroplacaceae(Malpighiaceae, Elatinaceae) clade's largely tropical distribution. Finally, Elatinaceae demonstrates long-term continental *in situ* diversification, which argues against recent dispersal as a universal explanation commonly invoked for aquatic plant distributions.

## Introduction

Aquatic angiosperms represent a diverse assemblage of species, which have arisen from terrestrial ancestors as many as 100 times [1]. The highly convergent and reduced nature of most aquatic plants has made it difficult to interpret their morphology in a phylogenetic context [2], but efforts during the last thirty years to clarify aquatic plant origins have resulted in tremendous progress. The biogeography of the vast majority of these aquatic plant clades, however, has yet to be investigated, resulting in uncertainty about whether aquatic plants show histories similar to their closest land-dwelling plant relatives. In their meta-analysis including a variety of phylogenetically diverse aquatic angiosperms, Les *et al*. [3] concluded i.) numerous aquatic plant clades are exceptionally widespread in their distribution (indeed, many are nearly cosmopolitan; see also Santamaría [4]) and ii.) aquatic angiosperms are generally too recent to be ascribed vicariant histories. These findings have been supported more recently by an investigation of the partially aquatic clade Haloragaceae [5]. Despite the ancient Eocene age of Haloragaceae, long-distance dispersal in the group was found to be much more recent (Miocene and younger) and strongly correlated with an aquatic habit.

To understand if more recent long-distance dispersal by aquatic plants applies universally, and if aquatic plants display divergent biogeographic histories relative to other land plants, a greater sampling of aquatic plant clades is required. Moreover, a phylogenetically informed sampling of aquatic taxa that includes their closest land plant relatives is important from a comparative standpoint. Along these lines, one aquatic plant clade that has received very little systematic or biogeographic attention is Elatinaceae. Elatinaceae is a bigeneric family of cosmopolitan aquatic herbs or shrubs; *Elatine* L. and *Bergia* L. have between them 35–50 species according to Tucker [6] and Leach [7]. *Elatine* contains about 25 species and is most diverse in the temperate zone of both hemispheres [6]: 12 species are found in Eurasia (three of which are also found in northern Africa), ten in North America, five to seven in South America (mostly in temperate to montane zones), two species in India and Malesia, and one in Australasia. *Bergia* also contains about 25 species and is most diverse in the Old World tropics, principally Africa and Australia [6, 7]: about 20 species occur in eastern and southern Africa, ten in Australia, five in southern Asia, two in Malesia, and three in the New World tropics (with one species, *Bergia texana* (Walp.) Seub., extending into temperate North America).

The closest relatives of Elatinaceae were uncertain during the early phase of the plant molecular systematics revolution [8]. However, over the course of several studies focused on resolving this uncertainty [8–10], most recently by sequencing plastomes across the order Malpighiales [11,12], the closest successive sister clades of Malpighiaceae, Centroplacaceae and Elatinaceae, have been confidently identified [herein referred to as the Centroplacaceae(Malpighiaceae, Elatinaceae) clade]. This increased phylogenetic resolution represents a unique opportunity to investigate the biogeography of Elatinaceae and their closest terrestrial relatives, members of the tropical families Malpighiaceae and Centroplacaceae. The juxtaposition of the woody tropical clades Centroplacaceae and Malpighiaceae versus the temperate (to montane tropical) aquatic clade Elatinaceae is interesting for two main reasons. The first, as we indicate above, is that there are surprisingly few biogeographic studies of aquatic angiosperms. Thus, a detailed phylogenetic and biogeographic study of the aquatic clade Elatinaceae will allow us for the first time to evaluate the taxonomy of the family in a phylogenetic framework, explore the degree of clade-level endemism within Elatinaceae, and investigate to what extent its aquatic ancestry can be explained by vicariance, or if rampant and recent dispersal predominates as has been hypothesized for aquatics more generally [3].

The second reason is that resolving the biogeographic history of Elatinaceae may help to clarify broader patterns of vicariant Gondwanan biogeography involving the tropics, especially the present-day landmasses of South America and Africa. One major way in which such disjunctions have been explained is via more recent dispersal across Laurasia, the northern supercontinent during the Tertiary, when northern latitudes were at least partially connected and supported tropical or subtropical vegetation [13–16]. The closest terrestrial relatives of Elatinaceae, Malpighiaceae, have provided evidence supporting the existence of this boreotropical superhighway [17–19], as have several other more recently published examples proposing dispersal involving the boreotropic [20–26]. Using densely sampled molecular phylogenies of Malpighiaceae combined with fossil evidence, seven disjunctions involving migrations from the New to the Old World have been inferred for this family during periods in the Eocene and Miocene when northern regions were tropical to subtropical. Subsequent cooling during these periods is hypothesized to have shunted these lineages from more northern localities into southern latitudes consistent with their present distribution [17–19]. This interpretation is further supported by excellent fossils of Malpighiaceae from northern localities in North America and Europe [27, 28] where they do not exist today.

**Fig 1:**
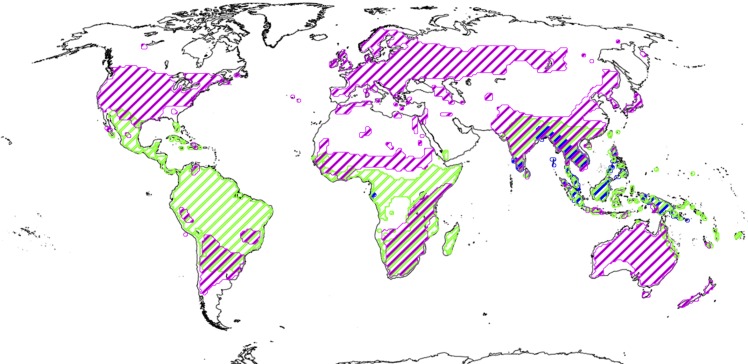
Distribution map of extant Centroplacaceae, Malpighiaceae, and Elatinaceae. Centroplacaceae demonstrate a disjoint distribution between western Africa and Southeast Asia (blue), while Malpighiaceae are predominantly located in tropical South America, North America, Africa, and Southeast Asia (green). Elatinaceae is cosmopolitan (magenta). Data are from the Angiosperm Phylogeny Website [33]; map is adapted from NASA Earth Observatory (http://earthobservatory.nasa.gov/).

Based on the fact that the early-diverging Malpighiaceae are found exclusively in the New World tropics, and are especially diverse in the ancient Guyana Shield region of northern South America, it has been assumed that Malpighiaceae originated in the New World tropics (Fig 1). Furthermore, it has been suggested that the origin of the family likely occurred during a time when Africa and South America (i.e., the supercontinent of western Gondwana) were sufficiently separate from each other that Gondwanan vicariance was unlikely. This hypothesis is supported by molecular divergence time estimates: the age of crown group Malpighiaceae has been inferred to be ∼75 Ma according to recent studies [28]. Results from other studies indicate slightly younger estimations for this age, ranging from ∼60–70 Ma [9, 17, 29], which is even less likely to support the vicariance hypothesis. Although paleoland configurations involving these landmasses are controversial, it appears that 110–80 Ma (summarized by Beaulieu *et al*. [30]) is a reasonable time span in which connections between these landmasses were severed, indicating that a strict western Gondwanan origin of crown group Malpighiaceae is unlikely. This hypothesis has never been properly tested, however, and is confounded by the fact that one of the earliest diverging clades within the family, the acridocarpoids, involves a migration into the Old World (predominantly involving Africa and Madagascar) [31]. The principal reason for this persistent impasse is that the closest relatives of Malpighiaceae, including Centroplacaceae, but particularly Elatinaceae, have remained unclear until very recently [8, 10–12, 32]. In particular, our lack of phylogenetic knowledge of Elatinaceae greatly hinders our resolution of this question. Given the wealth of data on Malpighiaceae, the time is right to address this question in a more comprehensive manner, by examining the extant evolutionary history of Elatinaceae.

## Materials and Methods

### Taxon and gene sampling

Our taxon and gene sampling for this study focused on the family Elatinaceae, which is poorly understood taxonomically. Here, we sampled 54 accessions of Elatinaceae including both of its genera, *Bergia* and *Elatine*, representing ∼75% of the currently recognized species in the family (30 species and 1 subspecies). We sampled broad taxonomic and biogeographic representatives of the family, including numerous individuals for single species to assess their monophyly. The genetic loci we sampled for this purpose–plastid *ndhF* and *matK*, and nuclear ITS–have previously been shown to be phylogenetically informative for resolving branches at various phylogenetic depths within Malpighiales [9,10, 31, 34–36]. Plastid *trnH*-*psbA* has been utilized to resolve more rapid radiations in other plant clades, and may be useful for resolving Elatinaceae. Amplification and sequencing protocols for *ndhF* followed Davis, Anderson, and Donoghue [36] using primers 5.5F and 10.2R, ITS followed Davis [35] using primers ITS4 and ITSLEU, *trnH*-*psbA* followed Ellison *et al*. [37] using primers trnH and psbA, and *matK* followed Cameron *et al*. [34] using primers trnK-2R and 842F. PCR amplicons were sequenced in both directions using Sanger sequencing at GENEWIZ, Inc. (Cambridge, MA; http://www.genewiz.com). Chromatograms were assembled into contiguous sequences and manually checked for accuracy using Geneious v6.1.6 (http://www.geneious.com/). All newly generated sequences with voucher information and GenBank accession numbers are reported in S1 Table. Sequence alignments are deposited in TreeBase (http://purl.org/phylo/treebase/phylows/study/TB2:S19273).

We assembled two data sets for phylogenetic analysis using different taxon sampling strategies. The first was targeted to infer relationships within Elatinaceae. The alignment of this data set included 847, 706, 433 and 1378 base pairs (bp) from *ndhF*, ITS, *trnH-psbA*, and *matK*, respectively. It included 54 accessions representing 30 species of Elatinaceae. Six Malpighiaceae and three Centroplacaceae outgroups from previous studies were used to root this phylogeny. Percentage of missing data in the first data set was 1.42% for *ndhF*, 19.7% for ITS, 42.4% for *trnH-psbA*, and 43.9% for *matK*. We found no evidence of concerns related to homoplasy, inversions, or a potential gene duplication as identified by Borsch & Quandt [38] and Meseguer *et al*. [25].

The second data set was used for the purposes of our broader biogeographic investigation of the (Centroplacaceae (Malpighiaceae, Elatinaceae)) clade. Here, we reduced our taxon sampling of Elatinaceae based on results from the Elatinaceae-centric analysis described above. Here, Elatinaceae species identified as monophyletic were reduced to single placeholders for each species identified. In cases where species were not monophyletic (i.e., *Elatine triandra* Schkuhr, *Elatine heterandra* H. Mason, and *Elatine chilensis* Gay), we included one placeholder representative of each of its constituent subclades. In addition, we also greatly expanded taxon sampling for Malpighiaceae. This family has been extensively sampled for previous phylogenetic and biogeographic analyses [10, 11, 17–19]. We subsampled from these published data to include the broadest range of morphological, phylogenetic, taxonomic, and biogeographic diversity in these families. The alignment of this data set included 896, 702, 1085 and 1396 bp from *ndhF*, ITS, *trnH-psbA*, and *matK*, respectively. This sampling included 34 Elatinaceae accessions representing 30 species and 1 subspecies, 165 Malpighiaceae accessions, and three Centroplacaceae species representing the broad phylogenetic diversity of this small family (two genera and six species) [10, 33]. The taxon sampling of Malpighiaceae included 165 species (all 68 currently recognized genera [33]) and was carefully selected to represent morphological, phylogenetic, and biogeographic diversity. *Peridiscus lucidus* Benth. (Peridiscaceae) was used as a more distant outgroup for rooting the tree, and was removed for the subsequent biogeographic analyses [8, 19].

### Phylogenetic analyses

Assembled sequences were aligned using MUSCLE v3.8.31 [39] and subsequently inspected manually. Gap positions with singleton insertions and deletions (INDELs) were deleted. For both data sets, all newly generated Elatinaceae sequences were concatenated with previously published data from Malpighiaceae and Centroplacaceae into a super matrix using Seaview v4.4.2 [40]. Phylogenetic reconstruction was conducted similarly for both data sets using maximum likelihood (ML) and Bayesian inference. ML analyses were conducted on the concatenated matrix using RAxML v7.2.8 [41]. The GTRGAMMA model was applied as determined by ModelTest v3.7 [42]. Optimal tree searches were conducted under the default settings. We also performed 1,000 rapid bootstrap replicates with the default settings to assess branch support. Bayesian analyses were implemented with the parallel version of BayesPhylogenies v2.0 [43] using the reversible-jump mixture model by Venditti *et al*. [44]. This approach allows the fitting of multiple models of sequence evolution to each character in an alignment without *a priori* partitioning, and was implemented by Xi *et al*. [11] to successfully resolve the deeper phylogenetic branching order within Malpighiales. Two independent Markov chain Monte Carlo (MCMC) analyses were performed, and the consistency of stationary-phase likelihood values and estimated parameter values was determined using Tracer v1.5 [45]. We ran each MCMC analysis for 10 million generations, sampling every 1,000 generations. Bayesian posterior probabilities (PPs) were determined by building a 50% majority-rule consensus tree from two MCMC analyses after discarding the 20% burn-in generations.

### Divergence time estimation

Divergence time estimates were conducted using treePL [46]. The input phylogeny included 202 representatives spanning Elatinaceae, Malpighiaceae, and Centroplacaceae. treePL uses the penalized likelihood framework developed by Sanderson [47], but combines the standard derivative-based optimization with a stochastic simulated annealing approach to overcome optimization challenges. The smoothing parameter was determined to be 0.1 using the random subsample and replicate cross-validation (RSRCV) approach [46]. RSRCV randomly samples terminal nodes, recalculates rates and dates with these nodes removed, and calculates the average error over the sampled nodes. In order to assess confidence intervals we then performed penalized likelihood across each of our previously generated 100 ML bootstrap trees. All of the results were summarized onto the optimal ML tree using TreeAnnotator v1.7.5 [48] and shown as 95% credible interval (CI) limits of the node heights.

Three Malpighiaceae fossils described in detail by Davis *et al*. [12, 18] were used as minimum age calibrations to constrain our phylogeny. In summary, a fossil species of *Tetrapterys* from the early Oligocene (33 Ma) of Hungary and Slovenia provided the minimum age constraint for the stem node of the *Tetrapterys* clades (S1 Fig node A). *Eoglandulosa warmanensis* from the Eocene Upper Claiborne formation of northwestern Tennessee (43 Ma) was used to constrain the stem node age for *Byrsonima* (Fig 3 node B). Finally, *Perisyncolporites pokornyi* provided a reliable stem node age (49 Ma) for the *stigmaphylloid* clade (S1 Fig node C). Reliably identified fossils of Elatinaceae and Centroplacaceae are unknown. The oldest Elatinaceae fossil discovered so far was a fossilized *Elatine alsinastrum* L. from middle to late Neogene (16–23 Ma) in Russia (http://www.fossil-cad.net/) [49]. We have not had a chance to study this fossil in detail. Regardless, it is unlikely to influence our conclusions because it is too recent for stem group *Elatine*. Finally, the root age of our phylogeny, which corresponds to the clade Centroplacaceae(Malpighiaceae, Elatinaceae), was fixed to 105 Ma based on secondary age constraints described by Xi *et al*. [11].

In addition to penalized likelihood using treePL, we also estimated divergence times using a Bayesian approach as implemented in BEAST v1.7.5 [50]. Our data set was partitioned by gene. Each locus was assigned a GTR+Γ model as determined by ModelTest v3.7 [42]. Priors for substitution models were set as default. The uncorrelated lognormal relaxed molecular clock model was applied, which allows rates to vary freely among the branches. Stepping-stone sampling [51] preferred the Yule process when compared to a birth-death process by a Bayes factor of 1.206 (which is not significant). Thus, we employed a Yule process for the tree prior and the optimal tree from treePL as starting tree. We fixed the Malpighiaceae+Elatinaceae clade to be monophyletic based on results from Xi *et al*. [11]. To obtain the posterior distribution of the estimated divergence times, three above mentioned fossil calibration points were applied as lognormal priors to constrain the node ages. The offset of each node constraint was set equal to the stratigraphic age of each fossil with a mean of 1.5 and standard deviation of 0.5. In addition, a universal prior for the mean root height ranging from 99.7–110.1 Ma was used based on Xi *et al*., [11]. Two independent Markov chain Monte Carlo (MCMC) analyses were conducted. We ran each MCMC analysis for 10 million generations, sampling every 1,000 generations. Log files were analyzed by Tracer v1.5. Tree files from the individual runs were combined using LogCombiner v1.7.5 from the BEAST package [50] after removing 2500 trees from each sample. The maximum-clade-credibility tree topology and mean node heights were calculated from the posterior distribution of the trees. Final summary trees were produced in TreeAnnotator v1.7.5.

### Reconstruction of biogeographic history

Our ancestral area reconstruction was conducted under both the dispersal-extinction-cladogenesis (DEC) model [41] and the modified dispersal–extinction–cladogenesis+J (DEC+J) model as implemented in the R package BioGeoBEARS v0.2.1 [52]. BioGeoBEARS incorporates information on the timing of both lineage divergences and the availability of connections between areas, enabling rates of dispersal, local extinction, and founder-event (in the DEC+J model) to be estimated by Bayesian inference. The DEC model in BioGeoBEARS is comparable to that implemented in Lagrange [53], except that BioGeoBEARS also allows the relative weighting of the different sorts of events to be made into free parameters rather than assigning equal probability to each event.

**Fig 2:**
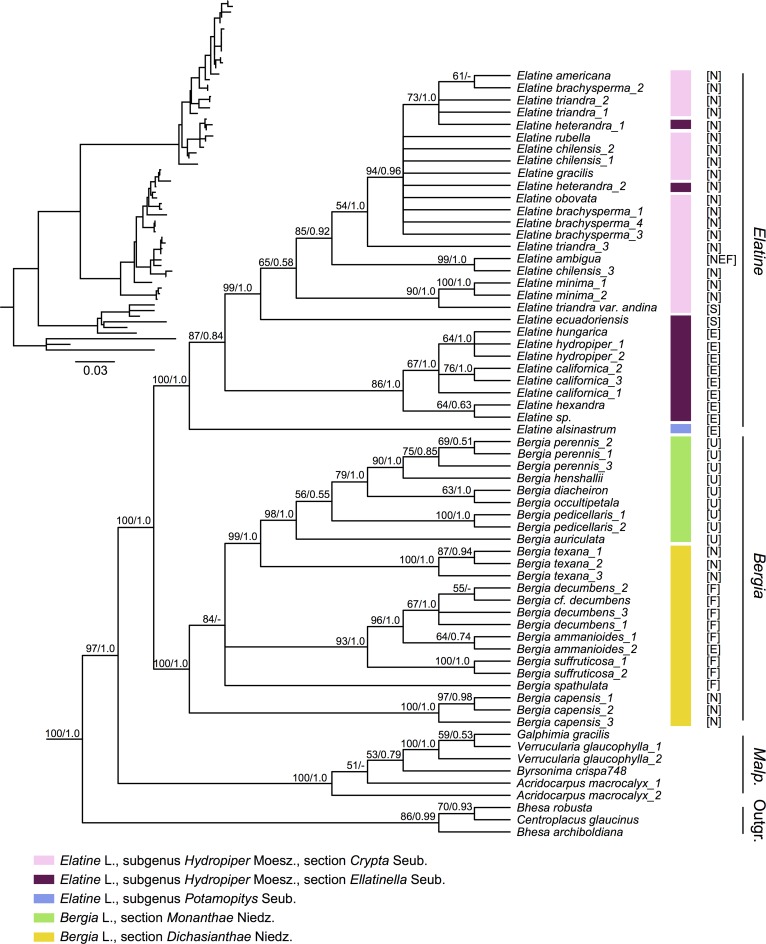
Fifty percent maximum likelihood (ML) majority-rule consensus tree of Elatinaceae based on the combined four-gene data set. Values above branches are ML bootstrap values (left) and Bayesian inference posterior probabilities (right). A hyphen indicates that the node is not present in a particular analysis. The relationship of Centroplacaceae(Malpighiaceae, Elatinaceae) was constrained for the purposes of our analyses. We assigned all of our sampled accessions into Niedenzu’s sectional grouping based on his original classification criteria [56]. Our placements of the Australian species in this classification were aided by the work of Leach [7]. Geographical distributions of species are abbreviated as follows: N = North and Central America, S = South America, E = Eurasia (Europe and Mainland Asia), F = Africa, U = Australia, Papua New Guinea, and the Pacific Islands, A = Insular Southeast Asia, including but not restricted to Malaysia, Indonesia, and the Philippines, M = Madagascar.

Biogeographic data for Elatinaceae were compiled from collection locality data (S1 Table), literature [6], and records from GBIF (http://data.gbif.org/welcome.htm). Data for Malpighiaceae and Centroplacaceae were from Davis *et al*. [19] and Anderson *et al*. [54]. Distribution ranges were categorized into seven areas (Fig 2), which largely followed those of Beaulieu *et al*. [30]. We additionally added Southeast Asia, which was distinguished from Eurasia to account for species solely distributed there (e.g., *Bhesa* spp. in Centroplacaceae). Categories of geographical distributions were abbreviated as follows: 1) N—North and Central America; 2) S—South America; 3) E—Eurasia (Europe and Mainland Asia); 4) F—Africa; 5) U—Australia, Papua New Guinea, and the Pacific Islands; 6) A—Insular Southeast Asia, including but not restricted to Malaysia, Indonesia, and the Philippines; 7) M–Madagascar. Widespread species were assigned to more than one area accordingly. *Bergia capensis* L. is known to be the most widely distributed species in the genus *Bergia* [6]. Despite its cosmopolitan distribution today, however, it is reported that *B*. *capensis* is only a recent adventive in areas outside of its native range of Africa and Southeast Asia [6, 55]. As a result, we coded *B*. *capensis* as endemic to Africa and Southeast Asia, although the specimen sampled for this study is from its introduced range in North and Central America. Finally, for the three non-monophyletic Elatinaceae species we identified (i.e., *E*. *triandra*, *E*. *heterandra*, and *E*. *chilensis–*see below), each placeholder was coded based on the distribution of its assigned species. This did not greatly influence our biogeographic analysis since all three species were from North America.

The underlying model of land configurations and dispersibility we implemented in Lagrange followed Mao *et al*. [57] (S2 Table). Each lineage was restricted to inhabit no more than two areas, reflecting the current distribution of the majority of extant species. We used five discrete time intervals to reflect major geological periods [57]: 105–70 Ma, 70–45 Ma, 45–30 Ma, 30–5 Ma, and 5–0 Ma. Dispersal probabilities ranged from 0.1 for well-separated areas to 1.0 for contiguous landmasses. The use of nonzero dispersal probabilities allowed for the possibility that lineages could occupy regions that are well separated today but were once connected. This also avoids the problem of the reducibility of Markov Chains [58].

In order to assess the impacts of different age estimates and varied range evolutionary model on the robustness of our conclusions, we conducted four biogeographic reconstructions using different data sources and range evolution models: 1) 100 dated bootstrap trees from treePL analyses as input phylogenies implementing the DEC model; 2) 100 dated bootstrap trees from treePL implementing the DEC+J model; 3) 100 randomly sampled trees from BEAST posterior distributions implementing the DEC model; and 4) 100 randomly sampled trees from BEAST posterior distributions implementing the DEC+J model. Results from these four analyses were summarized onto the optimum tree from treePL and maximum clade credibility tree from BEAST, respectively, using the script from PhyloWiki website (http://phylo.wikidot.com/biogeobears).

## Results

### Phylogenetic analyses

Our phylogeny from the Elatinaceae-centric analysis is largely well resolved (Fig 2). In this phylogeny, Centroplacaceae is fixed as outgroup [11,12] to Malpighiaceae plus Elatinaceae. Within Elatinaceae, *Bergia* and *Elatine* each form a well-supported clade (ML bootstrap percentage [BP] 100, Bayesian posterior probability [PP] 1.0). Within *Bergia*, the widespread species *B*. *capensis* is sister to rest of the genus (BP 100, PP 1.0). The remainder of *Bergia* is divided into a well-supported African clade, which is sister to a well supported, mostly Australian, clade (BP 84, PP< 0.5). Within *Elatine*, early diverging Eurasian species form a paraphyletic clade with a strongly supported clade of species inhabiting North and South America (BP 99, PP 1.0) embedded within. Species relationships within the North American clade are largely unresolved above 50 BP/0.50 PP. Nine species for which more than one accession was included are each identified as moderately to strongly supported clades (*Elatine minima* (Nutt.) Fisch. & C.A.Mey., *Elatine hydropiper* L., *Bergia ammanioides* (Roth) Wight & Arn., *Bergia capensis*, *Bergia decumbens* Planch. ex Harv., *Bergia pedicellaris* (F.Muell.) Benth., *Bergia perennis* (F.Muell.) Benth., *Bergia suffruticosa* Fenzl, *Bergia texana*). For a smaller subset of species resolution was less clear, but their monophyly could not be excluded as a possibility (*Elatine brachysperma* A.Gray, *Elatine californica* A.Gray). Three species of Elatinaceae were moderately to strongly supported as non-monophyletic. These include *E*. *triandra*, *E*. *heterandra*, and *E*. *chilensis*.

**Fig 3:**
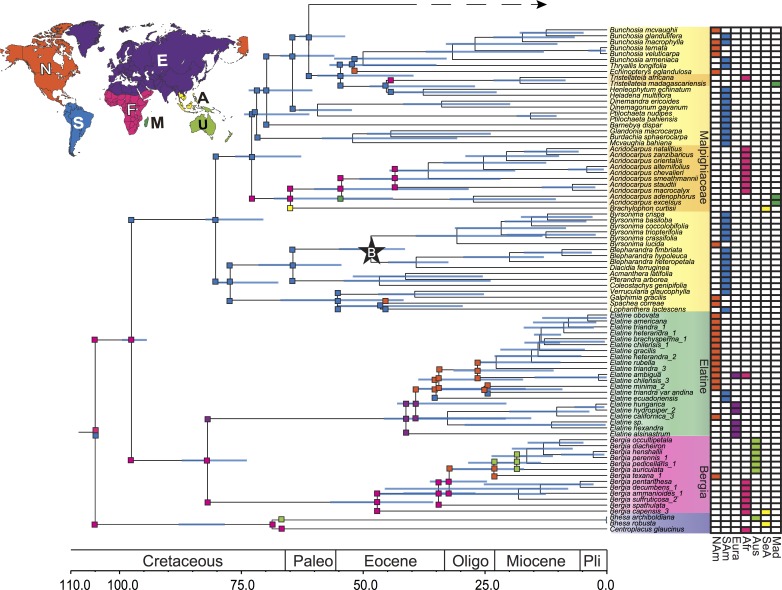
Biogeographic reconstructions of early diverging lineages of Centroplacaceae(Malpighiaceae, Elatinaceae) clade inferred using the DEC+J model. Ninety-five percent confidence intervals of the divergence time estimation using treePL shown in blue at each node. Fossil calibrations are marked by stars. Geographic distribution of each species is assigned to seven regions based on their collection localities and current distribution (colored boxes to the right). The range of each region is shown in the map, including North America (NAm, orange), South America (SAm, blue), Eurasia (Eura, purple), Africa (Afr, pink), Australia (Aus, light green), Southeast Asia (SeA, yellow), and Madagascar (Mad, dark green). Map is adapted from the Nature Earth (http://www.naturalearthdata.com/). The ancestral range reconstructions shown in color boxes at each node represent the scenarios with the highest marginal log-likelihood. Colored boxes at each branch represent geographic ranges immediately after a cladogenesis event. In the case where subsequent branches (or nodes) had the same information as the ancestral node, the boxes were suppressed for brevity. Continuous figure can be found in S1 Fig.

The broader phylogenetic analysis using our second data set produced a generally well-supported topology (Fig 3 and S1 Fig). Relationships within Elatinaceae using our phylogenetically guided reduced sampling described above are largely similar to those in Fig 2. In addition, the phylogeny of Malpighiaceae and Centroplacaceae was largely identical to previous results [10–12, 19, 34, 36].

### Divergence time estimation

The age of the Centroplacaceae(Malpighiaceae, Elatinaceae) clade was mid-Cretaceous (105 Ma), which was determined by the fixed age at the root. Based on penalized likelihood analyses from treePL, we estimated a Late Cretaceous origin of Malpighiaceae + Elatinaceae at 98 Ma (95%CI: 94.5–99.3 Ma). Crown group Malpighiaceae originated ∼80 Ma (95%CI: 70.6–82.4 Ma). The subsequent divergences along the backbone within crown group Malpighiaceae are estimated to occur from the Latest Cretaceous to the Early Eocene (51–71 Ma, 95%CI: 52.1–74.3 Ma).

Stem group Elatinaceae originated during the Late Cretaceous (82 Ma, 95%CI: 74.0–87.1 Ma). There was a long interval prior to the origin of the two extant genera, *Bergia* and *Elatine*. Crown group *Bergia* and *Elatine* originated during the early Eocene at 47 Ma (95%CI: 35.7–56.6 Ma) and 41 Ma (95%CI: 20.9–45.6 Ma), respectively. The divergence between *Bhesa* and *Centroplacus* within Centroplacaceae occurred in the Late Cretaceous, at 69 Ma (95%CI: 78.5–87.7 Ma).

The results from the BEAST analyses (S2 Fig, S3 Table) were largely comparable to the penalized likelihood analysis in treePL. The root age was estimated to be 109 Ma (95% HPD 106.1–110.1 Ma) and the origin of Malpighiaceae + Elatinaceae was inferred to be 107 Ma (95%HPD 103.9–110.0 Ma). Crown group Malpighiaceae originated in the Late Cretaceous at 88 Ma (95%HPD 82.4–93.2 Ma) and crown group Elatinaceae originated at 85 Ma (95%HPD 73.6–96.8 Ma). The optimum age estimates from Bayesian inference are generally older than those from penalized likelihood method with wider confidence intervals (S3 Table). However, these differences do not have a strong impact on our biogeographic estimations. Our main conclusions apply to all age estimation methods we implemented (S3–S6 Figs). For brevity, we focus our Discussion below on the results from the treePL analysis (Fig 3, S1 and S3 Figs).

### Biogeographic reconstructions

We focused our discussion on biogeographic reconstructions from the DEC+J model because it resulted in the highest maximum likelihood (S4 Table). Parameters of the DEC+J model were estimated as follows: d = 0.00196329, e = 1e-12, and j = 0.07943468. Results from Bayesian age estimation or using the DEC range evolution model can be found in the supplement (S4–S6 Figs).

Our seven-area biogeographic model estimated that the common ancestor of clade Centroplacaceae(Malpighiaceae, Elatinaceae) originated in the Southern Hemisphere regions of South America and Africa. The initial split (Fig 3 and S3 Fig) in this clade leads to the inheritance of Africa by stem group Malpighiaceae + Elatinaceae, with the South American portion of the range being lost immediately after the split event. After the origin of the common ancestor of Malpighiaceae + Elatinaceae in Africa, stem group Malpighiaceae shifted its range into South America. Following the origin and early diversification of crown group Malpighiaceae, there is evidence of multiple within-area divergence events prior to movement out of the region. This involved the early-diverging byrsonimoid clade in South America, around 77 Ma. Subsequently, the acridocarpoids assumed a solely African distribution with subsequent movement of this clade into Madagascar, while the banisterioids became restricted to South America. The remaining six movements from the New World to the Old World within the large banisterioid clade of Malpighiaceae mirror those published elsewhere [12, 17–19].

The reconstruction of Elatinaceae is more complex, involving within area diversification and multiple movements between areas. Stem group Elatinaceae originated in Africa. In the origin and diversification of crown group Elatinaceae, the scenario with the highest marginal probability estimates that the original split within crown group Elatinaceae gave rise to the inheritance of Africa in *Bergia* and a range shift into Eurasia in *Elatine*.

Within *Bergia*, the earliest split in the genus maintained an African distribution by the species *B*. *capensis* and its larger sister clade. Multiple subsequent *in situ* divergence events are estimated to take place during the early divergences within *Bergia*. Following this, a range shift is inferred from Africa to North America. The North American clade then underwent a split that gave rise to one clade inheriting its range in North America and the other clade moving to Australia, likely through long-distance dispersal. Multiple subsequent within-area divergence events are estimated after this initial movement into Australia.

The initial divergence within *Elatine* involved several early *in situ* diversifications in Eurasia, followed by a range shift into North America. Within the larger mostly North American subclade of *Elatine*, two independent range shifts into South America were represented by the species *Elatine ecuadorensis* Molau and *E*. *triandra* var. *andina*, respectively. The other North America clade underwent within-area diversification, which gave rise to the bulk of the genus.

## Discussion

### Phylogeny and systematics of Elatinaceae

Our analysis represents the first broad phylogenetic attempt to assess Niedenzu’s [56] taxonomic treatment of Elatinaceae (Fig 2). We identified several monophyletic groups in the family that correspond to his classification, including: *Bergia*, *Elatine*, *Bergia* section *Monanthae* Niedz., *Elatine* subgenus *Potamopitys* Seub, and *Elatine* subgenus *Hydropiper* Moesz. (S5 Table). Each of these subclades is supported by morphology, and in some cases also biogeography. *Bergia* species are terrestrial or partially aquatic herbs to subshrubs with persistent glandular pubescence, 5-merous flowers, and an ovoid capsule. *Elatine* species are fully aquatic glabrous herbs, with 2-4-merous flowers, and globose capsules [6]. *Elatine* is further divided into two subgenera. Subgenus *Potamopitys*, which includes the single species *E*. *alsinastrum*, is characterized by its whorled leaves. Species in subgenus *Hydropiper*, in contrast, are distinguished by their opposite leaves. *Bergia* is divided into two sections based on the nature of their inflorescences and biogeography, only one of which is monophyletic. Section *Monanthae* includes species mainly distributed in Australia and possessing solitary axillary flowers.

We also identified several higher level taxonomic designations *sensu* Niedenzu that are not monophyletic, including: *Bergia* section *Dichasianthae* Niedz, *Elatine* section *Elatinella* Seub., and *Elatine* section *Crypta* Seub. Species of *Bergia* section *Dichasianthae*, which are mainly distributed in Africa and North America, possess more than one flower borne in axillary dichasia. These species form a paraphyletic assemblage of clades that are successively sister to the monophyletic section *Monanthae* described above. In the cases of *Elatine* sections *Elatinella* and *Crypta*, diagnostic features used by Niedenzu are problematic. Our phylogeny placed all North American *Elatine* species in one clade, which largely corresponds to Niedenzu’s section *Crypta*. The exception is *E*. *heterandra*: although we place it as a nested member of this North American subclade with other members of section *Crypta*, Niedenzu instead assigned it to section *Elatinella*. Species in section *Crypta* are characterized by having two or three stamens per flower, while species in section *Elatinella* have six to eight per flower. *E*. *heterandra*, however, may have either three or six stamens [59], thus raising doubt of the utility of stamen number to delimit taxa within *Elatine*.

Finally, we also identified several species that are non-monophyletic, including *E*. *heterandra*, *E*. *triandra*, and *E*. *chilensis* (Fig 2). Different accessions of these species formed well-supported, non-monophyletic subclades. Species delimitation within Elatinaceae is beyond the scope of our study, but brings to light the need for broader taxonomic and monographic revision in the family.

### Western Gondwanan vicariance of pantropical Centroplacaceae(Malpighiaceae, Elatinaceae)

**Fig 4:**
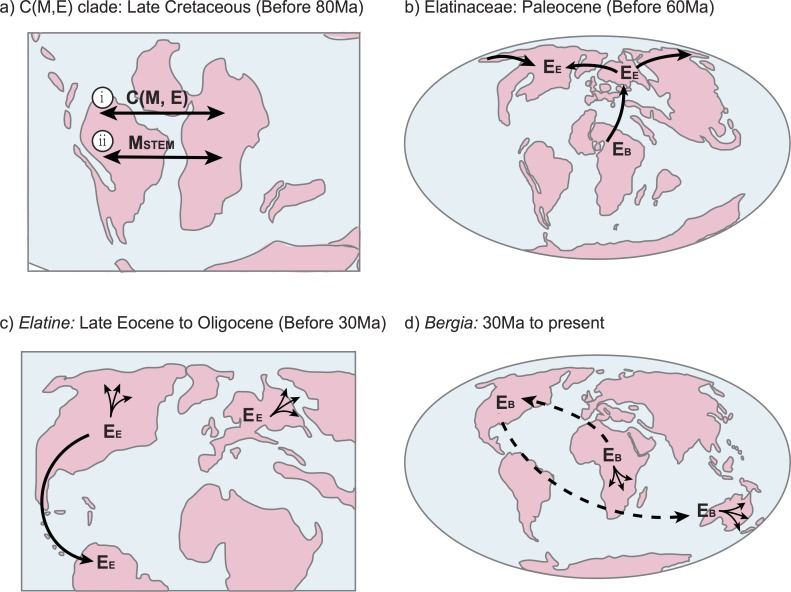
Spatiotemporal evolution of the Centroplacaceae(Malpighiaceae, Elatinaceae) clade. a) Early evolutionary history of Centroplacaceae [C] (Malpighiaceae [M], Elatinaceae [E]) reflecting hypothesized western Gondwanan vicariance scenarios involving South America and Africa, including: i.) the split of Centroplacaceae(Malpighiaceae, Elatinaceae); ii.) the range shift of stem group Malpighiaceae (MSTEM) from Africa into South America. b) Initial divergence events within Elatinaceae: the initial split within crown group Elatinaceae coincided with African inheritance by *Bergia* (EB) and a range shift to Eurasia by *Elatine* (EE). *Elatine* then spread into North America via the North Atlantic land bridge or Beringia. c) Movement and diversification within *Elatine*: after the early occupation by *Elatine* of North America and Eurasia, the lineages split in the late Eocene. This split is hypothesized to result from climate change or reduced paleoland availability. The North American lineage both diversified locally (spread arrows) and migrated into South America (black arrow), while the European lineages diversified independently (spread arrows). d) Dispersal of *Bergia* into North America and Australia: after the early diversification of *Bergia* within Africa, a split occurred between these landmasses in the early Oligocene. This split gave rise to a wholly African lineage that underwent recent *in situ* diversification (spread arrows) and a hypothesized migration into North America. One lineage from the largely North American clade then moved into Australia. These two migrations possibly involve long-distance dispersal (dashed line). Paleogeographical maps are modified from [56], which is based on C.R. Scotese's Paleomap project (http://www.scotese.com/earth.htm).

Given that the origin of numerous Malpighiales clades extend reasonably deep into the Cretaceous [9, 11], and because the most recent common ancestor of Centroplacaceae(Malpighiaceae, Elatinaceae) appears to have been present in the Southern Hemisphere (Fig 3), we specifically addressed whether any of the between-area disjunctions involving these clades were consistent with Gondwanan vicariance. Based on our biogeographic estimates, the likeliest scenario is that the common ancestor of Centroplacaceae(Malpighiaceae, Elatinaceae) was widely distributed across South America and Africa (i.e., former members of western Gondwana; Fig 3). The split at this widespread ancestor gave rise to two African lineages, stem group Centroplacaceae and stem group (Malpighiaceae, Elatinaceae), which have lost their range distribution in South America immediately after this split. From a paleoland perspective, this disjunction involving Africa and South America suggests ancient vicariance involving western Gondwana. Furthermore, such a vicariant event is feasible from an age standpoint. The timing of direct overland connections between present day South America and Africa remains unclear, but it appears they may have been in existence until between ∼110–80 Ma [30]. Despite our fixed root age of 105 Ma using penalized likelihood, the Bayesian inference accommodating uncertainty in root age produced a very similar estimate (109 Ma; 95%HPD 106.1–110.1 Ma, S3 Fig, S3 Table). Thus, our results suggest that this initial split likely occurred during the Late Cretaceous indicating that the Centroplacaceae(Malpighiaceae, Elatinaceae) disjunction involving South America and Africa possibly occurred during a time when these two continents were connected or in very close proximity to each other, forming the supercontinent western Gondwana (Fig 4A). Biogeographic reconstructions involving Australia do not appear in our phylogeny until more recently, and involve crown group Centroplacaceae. According to our reconstructions, the initial split within crown group Centroplacaceae probably leads to the occupation of Australia and nearby Pacific islands by *Bhesa* during the late Cretaceous. The time of this movement is estimated to be 69 Ma (95% CI: 78.5–87.7 Ma). The age and areas coincide with the possibility of a southern hemisphere migration involving Indo-Madagascar and Australia/Antarctica through the Kerguelen Plateau. Such a migration has been proposed most famously in ratite birds [60], and would also be supported here by the temperate climates inhabited by many extant Elatinaceae species. It is worth noting, however, that additional biogeographic sampling in *Bhesa* to include its range in southern China and southern India may increase the possibility that a boreotropical route may better explain their migration from Africa. Lastly, neither of these scenarios may apply in which case this range shift may instead be attributed to dispersal.

Crown group (Malpighiaceae, Elatinaceae) is similarly ancient and retained its distribution in Africa by the Late Cretaceous, 98 Ma (95% CI: 94.5–99.3 Ma). However, a range shift from Africa to South America is estimated by one of these two descendant subclades, stem group Malpighiaceae. Given the age estimates of stem group Malpighiaceae (98 Ma, 95% CI: 94.5–99.3 Ma), this migration may have similarly been facilitated by more direct overland connections between South America and Africa. Thus, the disjunct distribution of stem group Malpighiaceae and stem group Elatinaceae may also be attributed to Gondwanan vicariance. Stem group Elatinaceae inherited its African range (98 Ma, 95% CI: 94.5–99.3 Ma) and then expanded into Eurasia after the initial split of its crown group (82 Ma, 95% CI: 74.0–87.1 Ma). Given the long time duration and very large geographic areas involved (Africa and Eurasia), the nature of their migration from Africa into Eurasia is unclear. One possibility is that they utilized dispersal corridors involving parts of present-day western Europe (Fig 4B). Such a scenario has been invoked for the southward migration of the acridocarpoids (Malpighiaceae) from boreotropical regions into Africa and Madagascar [35].

Additionally, we confirmed a South American origin of crown group Malpighiaceae. The initial split within the family involves the byrsonimoid clade, which today includes only New World endemics, plus a more broadly distributed lineage, which today includes the Old World acridocarpoids and the remainder of the mostly New World Malpighiaceae. Previous biogeographical hypotheses indicated that the initial movement of the acridocarpoids from the New to the Old World was likely via Laurasian boreotropical migrations [17, 18]. Based on our estimates on the timing of this split (73 Ma, 95%CI: 62.8–72.1 Ma), a direct overland connection between South America and Africa during this time is not likely and thus more supportive of the earlier boreotropical hypothesis. Such boreotropical route was also hypothesized to facilitate the dispersal of the ancient tropical clusioid clade (Malpighiales) during the Eocene [61]. Scattered island chains in the South Atlantic [62] may also have served as a route of migration to the New World. These oceanic islands may have persisted until as recently as the Middle Eocene and have been hypothesized to have facilitated the migration of passerine birds [63], platyrrhine primates [62], and caviomorph rodents [62]. The remaining six cases of migration from the New to the Old World fall within the banisterioid clade, and are well outside of these more ancient timeframes, which supports previous biogeographical conclusions involving the majority of these Old/New World disjunctions.

In summary, we have identified two ancient biogeographic events potentially involving the formerly connected Gondwanan landmasses of Africa and South America: i.) the split of Centroplacaceae(Malpighiaceae, Elatinaceae); ii.) the range shift of stem group Malpighiaceae from Africa into South America. These results support previous findings that detecting such ancient biogeographic events will likely rely on investigating larger and older clades, many of which involve groups of traditionally recognized families or orders [30, 64, 65]. Our discoveries also confirm inferences on the biogeographic origins of Malpighiaceae, and are one of only a few phylogenetic studies to implicate such ancient Gondwanan biogeographic patterns involving extant angiosperm lineages. This is significant especially given the clade's largely tropical distribution, for which there are few clear examples of Gondwanan angiosperm histories. In light of our findings, we hypothesize that there are likely numerous such Gondwanan vicariant events remaining to be uncovered in the order Malpighiales, owing to its widespread, mostly pantropical, distribution and ancient divergence times of the families within the order. Most families or groups of families in Malpighiales significantly pre-date the K/T boundary [9, 11, 12]. Future studies investigating these patterns will require sufficiently broad taxonomic and biogeographic sampling within Malpighiales clades to address these deeper patterns of diversification across the order.

### Biogeography of Elatinaceae

Our results also allow us to explore the biogeographic history of an ancient aquatic clade. As we indicate above, crown group Elatinaceae is 82 Ma (95% CI: 74.0–87.1 Ma)], and is inferred to have originated in Africa. The first split within the family between stem group *Elatine* and *Bergia* involved a range shift by *Elatine* from Africa into Eurasia (Fig 4B); *Bergia*, in contrast, maintained its home range in Africa apparently for tens of millions of years (95% CI: 74.0–87.1 Ma, stem group age; 95% CI: 35.7–56.6 Ma, crown group age). We address the biogeographic histories of *Elatine* and *Bergia* separately in the subsequent discussion.

The timing of the range shift of stem group *Elatine* from Africa into Eurasia is estimated to be 82 Ma (95% CI: 74.0–87.1 Ma) possibly through present-day western Europe as discussed above. At 39 Ma (95% CI: 20.7–42.9 Ma) we see a clade split and range shift of one clade from Eurasia to North America. During this time, dispersal corridors via the North Atlantic or Beringia were likely available (Fig 4B), and may have facilitated this hypothesized expansion across the northern hemisphere [66, 67]. The divergence time estimates of this biogeographic event, if involving a North Atlantic connection, broadly coincides with the deterioration of tropical climates and reduced paleoland availability across this region [14, 16]. The two descendant lineages resulting from this northern hemisphere disjunction gave rise to i.) a Eurasian subclade, which underwent extensive *in situ* diversification, and ii.) a subclade that expanded southward from North America into South America (Fig 4C). The estimate of this southward expansion is 35 Ma (95% CI: 16.8–39.6 Ma; crown group age). This may have occurred at a time when Central America was becoming increasingly porous to plant migrations. Recent investigations by Willis *et al*. [68], in particular, show a significant transition in the rate of migration across this region beginning in the late Oligocene, which supports a model of increasing land availability for such migrations to have occurred starting from this time period. Following subsequent *in situ* diversification involving South America and North America, we see another split, which gives rise to a large North American radiation of *Elatine*, and a clade that maintains its broader ancestral range of South America and North America.

Crown group *Bergia* originated in Africa and experienced *in situ* diversification. A subsequent split occurred in the early Oligocene (32 Ma, 95% CI: 24.7–36.2 Ma) giving rise to a wholly African radiation and another clade that migrated to North America (Fig 4D). Shortly after this migration, the North American clade split into two clades (27 Ma, 95% CI: 11.1–31.3 Ma), one represented by the species *B*. *texana* retaining the North American range, the other migrated to Australia. The Australia lineage then gave rise to a mostly Australian radiation, where *Bergia* species exist in abundance today. Given the estimated age and areas involved, these two migrations within *Bergia* are likely to be attributed to long-distance dispersal (Fig 4D), since there were no obvious land connections between Africa, North America, and Australia.

These findings lend insight into the biogeography of Elatinaceae, but also, more broadly, into the biogeography of aquatic angiosperms. In contrast to Les *et al*. [3] who concluded that aquatic plants show recent dispersal events, we find that this does not apply universally to the biogeographic histories of aquatic clades. Elatinaceae, while demonstrating likely evidence of possible dispersal, do not appear to be especially dispersal-prone relative to their closest terrestrial relatives. Moreover, we see a high degree of long-term *in situ* continental diversification in Elatinaceae, which is less supportive of the conclusion that dispersal is a universal explanation for the distribution in this aquatic clade. If dispersal were a main factor, we would not expect to see the high degree of endemicity in Elatinaceae, including the restriction of stem group *Bergia* to Africa for millions of years as well as the independent *in situ* radiations of *Elatine* in North America and Eurasia, and *Bergia* in Australia and Africa. Although we agree that dispersal is likely an important factor in aquatic angiosperms, as it is for many other plant clades, our focus on aquatic lineages of more ancient ancestry has expanded the view that all aquatic distributions are attributable to dispersal.

### Adaptation of aquatic plants to temperate regions

One class of dispersal event in the biogeographic history of Elatinaceae is of great interest and deserves special attention. The movement from tropical to more temperate regions that characterize extant Elatinaceae is unusual among Malpighiales–excursions into the temperate zones within this largely tropical order are restricted to a relatively small number of clades, otherwise occurring in Euphorbiaceae, Hypericaceae, Chrysobalanaceae, Linaceae, Passifloraceae, Picrodendraceae, Phyllanthaceae, Podostemaceae, Salicaceae, and Violaceae. Although we have not explored the precise spatial and temporal context for when this occurred, it is likely that this transition took place within Elatinaceae and was facilitated by some degree of adaptation to temperate environments. In particular, the wholly aquatic genus *Elatine* occurs solely in temperate and tropical montane regions. Although *Elatine* has survived and expanded its range through northern areas, the clade's movement out of Africa would have taken place when these areas were likely dominated by more tropical elements, similar to the history hypothesized for the Hypericaceae by Meseguer *et al*. [25, 26]. Today, *Elatine* species in the northern hemisphere inhabit temperate environments, suggesting that they evolved temperate adaptations after moving north. An alternative is that they evolved such adaptations in mountainous regions of Africa, which were more temperate in nature, and then dispersed northward.

What then is the mechanistic basis underpinning the movements and persistence of *Elatine* outside the tropics? Here, it is tempting to speculate that *Elatine* may have some evolutionary advantage in moving between temperate and tropical environments. The usual dispersal agent invoked is a well-traveled but poorly preened waterfowl, and the small, sticky seeds of Elatinaceae are good candidates for such dispersals. However, for dispersal to succeed, any introduced plant must still persist in its new environment.

We suggest that one physiological cause for an aquatic survival advantage could be a simple buffering effect offered by underwater living. Because water holds heat during cold nights, plants beneath the water would have a reduced chance of tissue damage from freezing. This might leave aquatic plants more likely than terrestrial plants to become established in temperate zones. Further, *Elatine* employs cleistogamy when immersed, so that a single colonizing plant could easily reproduce. Perhaps most importantly, *Elatine* tends to be ephemeral in flooded areas or seasonal pools, and to have persistent seeds, which are durable in both submerged and dry conditions. Thus, its life cycle would be synchronized with that of similar plants, emerging in ideal conditions for growth and reproduction. These factors could lead to interbreeding of successive introductions, increasing genetic diversity and leading to rapid adaptation and expansion within their new range [14]. Such advantage for aquatics in transition to temperate living could partially explain the noted phenomenon of hydrophytes being very widespread compared to terrestrial plants (reviewed by Les *et al*. [3]).

This hypothesis offers the potential for very interesting future work inferring ancestral climate states based on extant Elatinaceae and correlating climate with their geographical history. The movements of Elatinaceae in the Americas and Europe have been unexpectedly complex, and evaluating the influence of climate will allow a deeper understanding of their radiations and migrations.

## Supporting Information

S1 Fig Biogeographic reconstructions of core Malpighiaceae clade inferred using the DEC+J model. Ninety-five percent confidence intervals of the divergence time estimation using treePL shown in blue at each node. Fossil calibrations are marked by stars. Geographic distribution of each species is assigned to seven regions based on their collection localities and current distribution (colored boxes to the right). The range of each region is shown in the map, including North America (NAm, orange), South America (SAm, blue), Eurasia (Eura, purple), Africa (Afr, pink), Australia (Aus, light green), Southeast Asia (SeA, yellow), and Madagascar (Mad, dark green). The ancestral range reconstructions shown in color boxes at each node represent the scenarios with the highest marginal log-likelihood. Colored boxes at each branch represent geographic ranges immediately after a cladogenesis event. In the case where subsequent branches (or nodes) had the same information as the ancestral node, the boxes were suppressed for brevity. Biogeographic reconstructions of basal clade Centroplacaceae(Malpighiaceae, Elatinaceae) can be found in Fig 3.
(TIFF)

S2 Fig Bayesian age estimates of Centroplacaceae(Malpighiaceae, Elatinaceae) inferred using BEAST. 95% HPD for each node age is shown with blue bar at nodes.
(TIFF)

S3 Fig Biogeographic reconstructions of Centroplacaceae(Malpighiaceae, Elatinaceae) inferred under the DEC+J model using nodal ages estimated from treePL. Results estimated from 100 bootstrap trees are summarized onto the optimum tree from ML analysis. Pie charts at nodes show marginal log-likelihood of each geographical range scenario. A maximum of two areas is allowed for each ancestral species. Areas are represented as follows: N = North and Central America, S = South America, E = Eurasia (Europe and Mainland Asia), F = Africa, U = Australia, Papua New Guinea, and the Pacific Islands, A = Insular Southeast Asia, including but not restricted to Malaysia, Indonesia, and the Philippines, M = Madagascar.
(TIFF)

S4 Fig Biogeographic reconstructions of Centroplacaceae(Malpighiaceae, Elatinaceae) inferred under the DEC+J model using nodal ages estimated from BEAST. Results estimated from 100 trees sampled from the posterior distribution are summarized onto the optimum tree from the ML analysis. Pie charts at nodes show marginal log-likelihood of each geographical range scenarios. A maximum of two areas is allowed for each ancestral species. Areas are represented as follows: N = North and Central America, S = South America, E = Eurasia (Europe and Mainland Asia), F = Africa, U = Australia, Papua New Guinea, and the Pacific Islands, A = Insular Southeast Asia, including but not restricted to Malaysia, Indonesia, and the Philippines, M = Madagascar.
(TIFF)

S5 Fig Biogeographic reconstructions of Centroplacaceae(Malpighiaceae, Elatinaceae) inferred under the DEC model using nodal ages estimated from treePL. Results estimated from 100 bootstrap trees are summarized onto the optimum tree from the ML analysis. Pie charts at nodes show marginal log-likelihood of each geographical range scenarios. A maximum of two areas is allowed for each ancestral species. Areas are represented as follows: N = North and Central America, S = South America, E = Eurasia (Europe and Mainland Asia), F = Africa, U = Australia, Papua New Guinea, and the Pacific Islands, A = Insular Southeast Asia, including but not restricted to Malaysia, Indonesia, and the Philippines, M = Madagascar.
(TIFF)

S6 Fig Biogeographic reconstructions of Centroplacaceae(Malpighiaceae, Elatinaceae) inferred under the DEC model using nodal ages from BEAST. Results estimated from 100 trees sampled from the posterior distribution are summarized onto the optimum tree from ML analysis. Pie charts at nodes show marginal log-likelihood of each geographical range scenarios. A maximum of two areas is allowed for each ancestral species. Areas are represented as follows: N = North and Central America, S = South America, E = Eurasia (Europe and Mainland Asia), F = Africa, U = Australia, Papua New Guinea, and the Pacific Islands, A = Insular Southeast Asia, including but not restricted to Malaysia, Indonesia, and the Philippines, M = Madagascar.
(TIFF)

S1 Table Taxa sequenced with distribution, voucher information, and GenBank accession numbers. “Distr.” corresponds to ranges of species applied to biogeographic analyses: N = North and Central America, S = South America, E = Eurasia (Europe and Mainland Asia), F = Africa, U = Australia, Papua New Guinea, and the Pacific Islands. “-” = data unavailable.
(DOCX)

S2 Table Time-stratified dispersal rate matrices used in the BioGeoBEARS analysis derived from [57]. Categories of geographical distributions were abbreviated as follows: 1) N—North and Central America; 2) S—South America; 3) E—Eurasia; 4) F—Africa; 5) U—Australia, Papua New Guinea, and the Pacific Islands; 6) A—Insular Southeast Asia, including but not restricted to Malaysia, Indonesia, and the Philippines; 7) M–Madagascar.
(DOCX)

S3 Table Age estimations for early divergence events within (Centroplacaceae(Malpighiaceae,Elatinaceae)) clade using treePL and BEAST. (DOCX)

S4 Table Estimated likelihood for biogeographic reconstruction with input phylogenies from treePL/BEAST under DEC/DEC+J model. (DOCX)

S5 Table Comparison of the phylogenetic results from this study with the classification of Elatinaceae sensu Niedenzu (1925); * denotes a monotypic taxon. (DOCX)
