# Supplementary figures and images for: Phylogeny of Elatinaceae and the Tropical Gondwanan Origin of the Centroplacaceae(Malpighiaceae, Elatinaceae) Clade
Source: PLoS One. 2016 Sep 29;11(9):e0161881. doi: 10.1371/journal.pone.0161881 (PMC5042423; doi:10.1371/journal.pone.0161881)

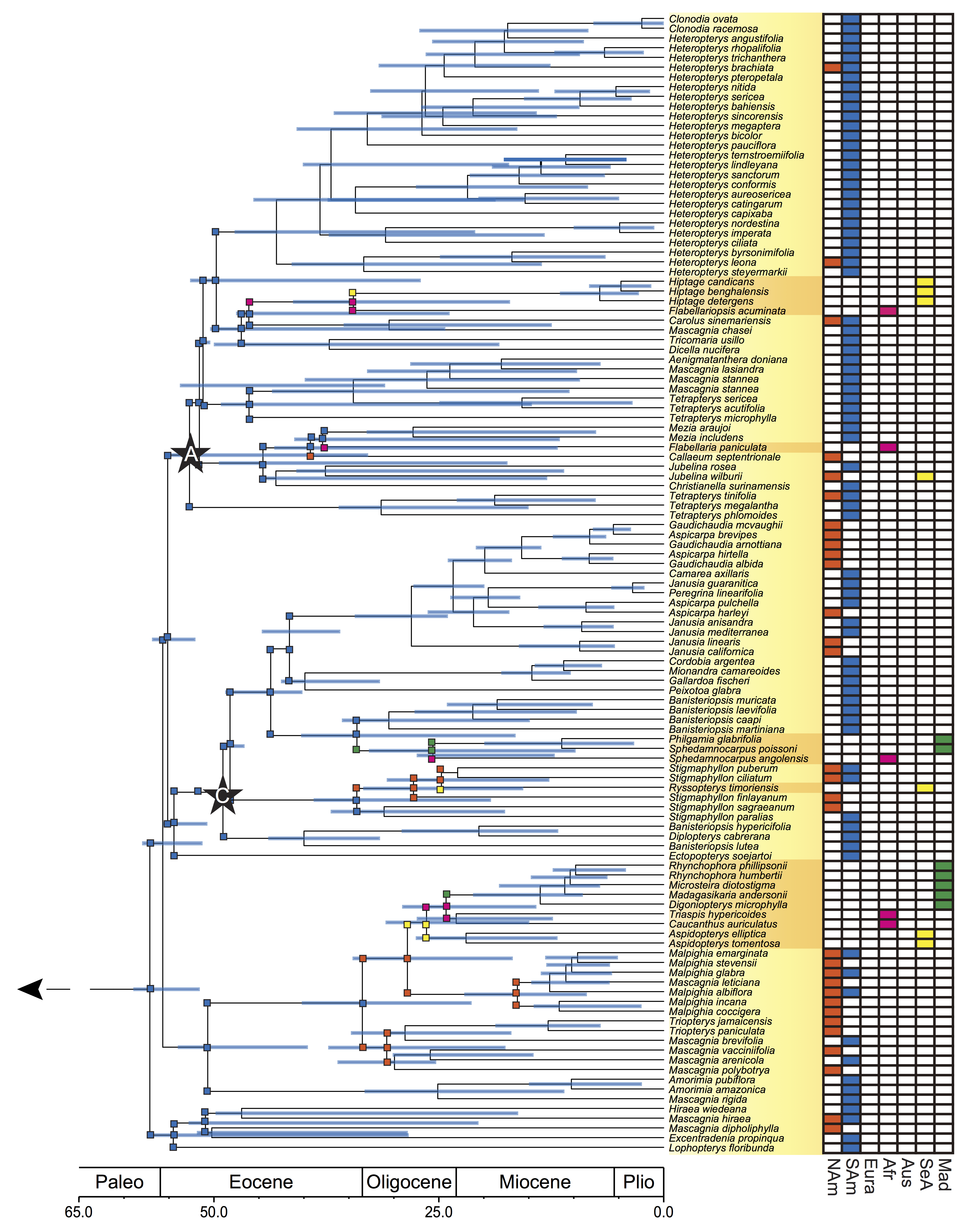

Supplement: S1 Fig — Ninety-five percent confidence intervals of the divergence time estimation using treePL shown in blue at each node. Fossil calibrations are marked by stars. Geographic distribution of each species is assigned to seven regions based on their collection localities and current distribution (colored boxes to the right). The range of each region is shown in the map, including North America (NAm, orange), South America (SAm, blue), Eurasia (Eura, purple), Africa (Afr, pink), Australia (Aus, light green), Southeast Asia (SeA, yellow), and Madagascar (Mad, dark green). The ancestral range reconstructions shown in color boxes at each node represent the scenarios with the highest marginal log-likelihood. Colored boxes at each branch represent geographic ranges immediately after a cladogenesis event. In the case where subsequent branches (or nodes) had the same information as the ancestral node, the boxes were suppressed for brevity. Biogeographic reconstructions of basal clade Centroplacaceae(Malpighiaceae, Elatinaceae) can be found in Fig 3. (TIFF) [file pone.0161881.s001.tiff]

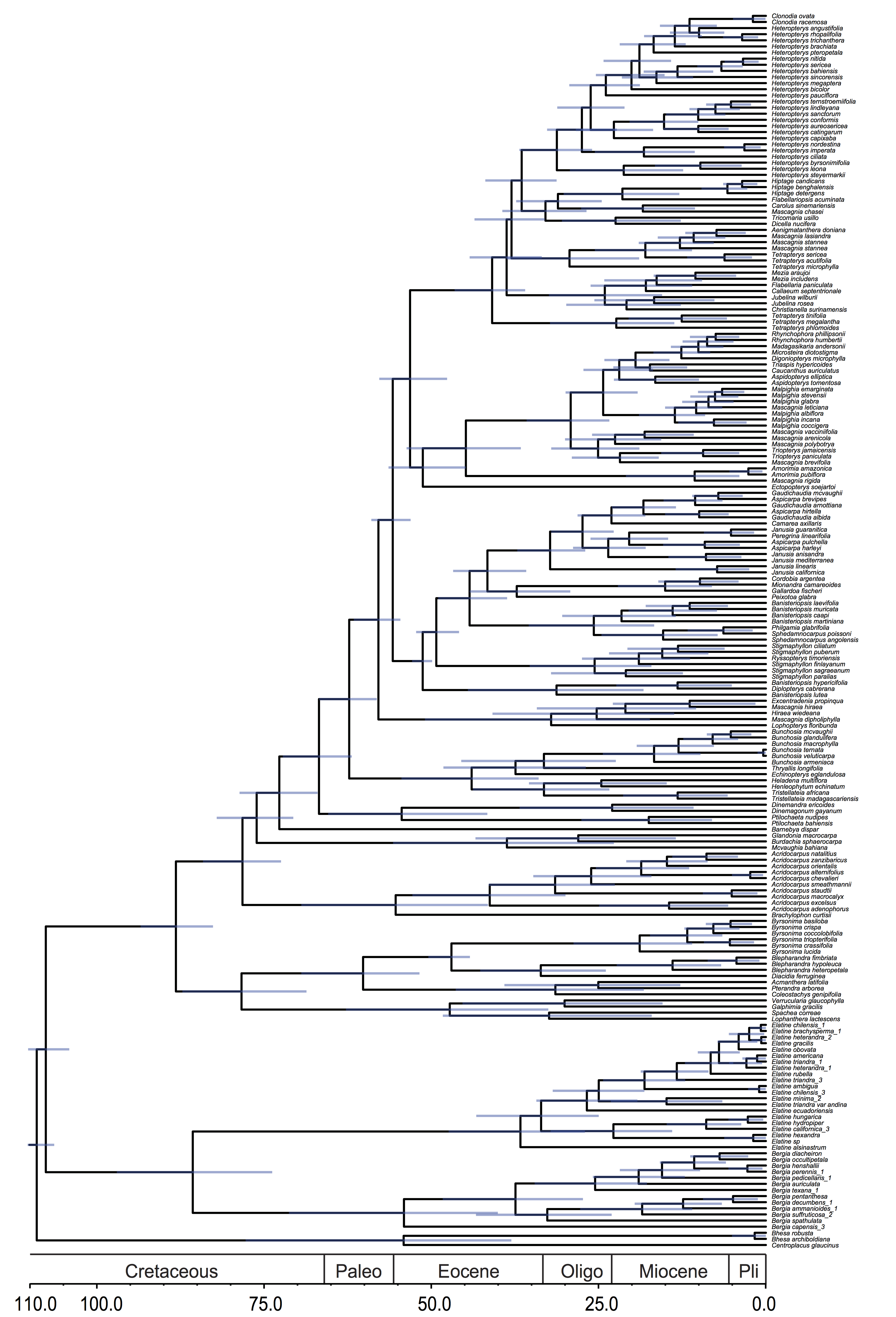

Supplement: S2 Fig — 95% HPD for each node age is shown with blue bar at nodes. (TIFF) [file pone.0161881.s002.tiff]

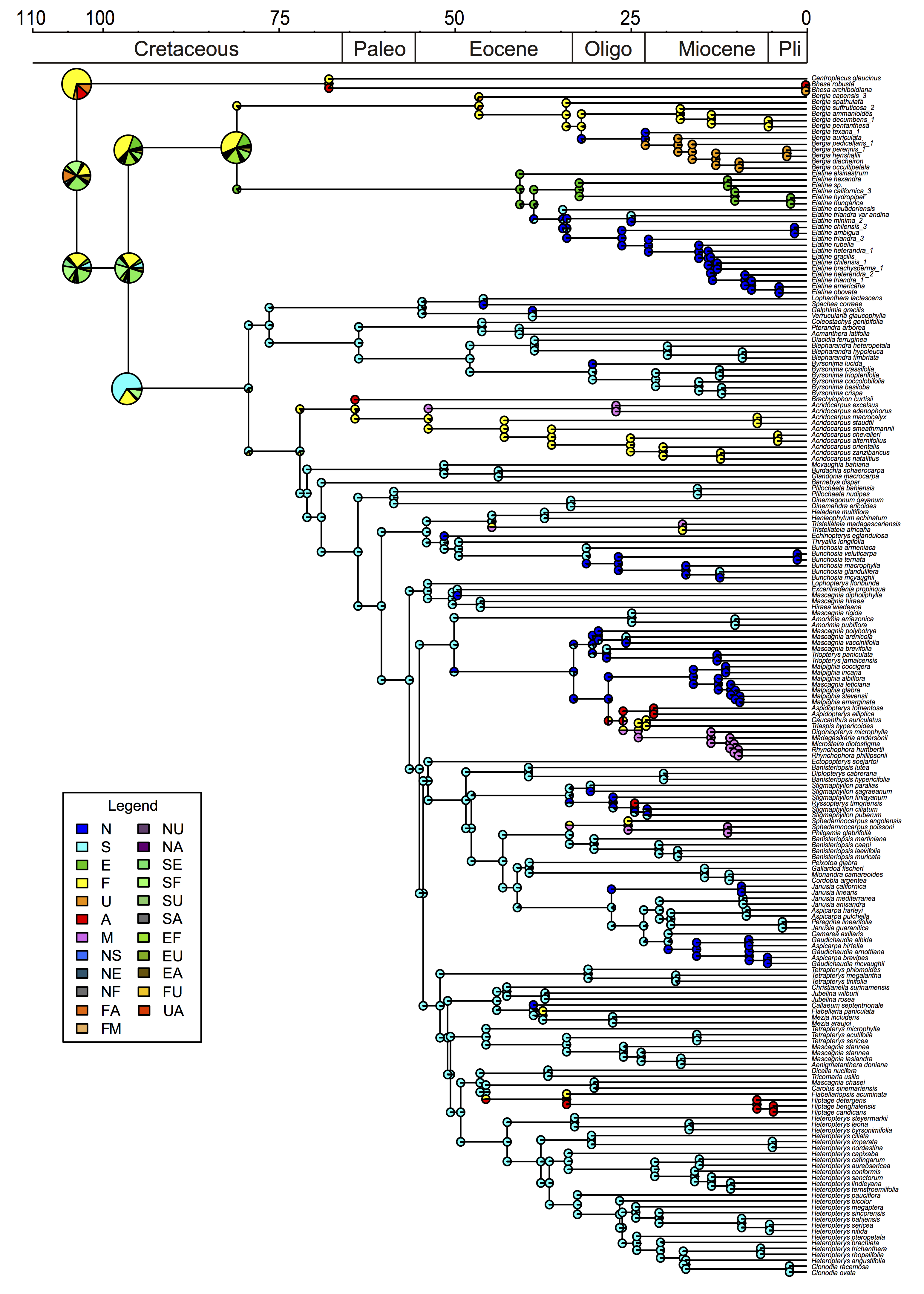

Supplement: S3 Fig — Results estimated from 100 bootstrap trees are summarized onto the optimum tree from ML analysis. Pie charts at nodes show marginal log-likelihood of each geographical range scenario. A maximum of two areas is allowed for each ancestral species. Areas are represented as follows: N = North and Central America, S = South America, E = Eurasia (Europe and Mainland Asia), F = Africa, U = Australia, Papua New Guinea, and the Pacific Islands, A = Insular Southeast Asia, including but not restricted to Malaysia, Indonesia, and the Philippines, M = Madagascar. (TIFF) [file pone.0161881.s003.tiff]

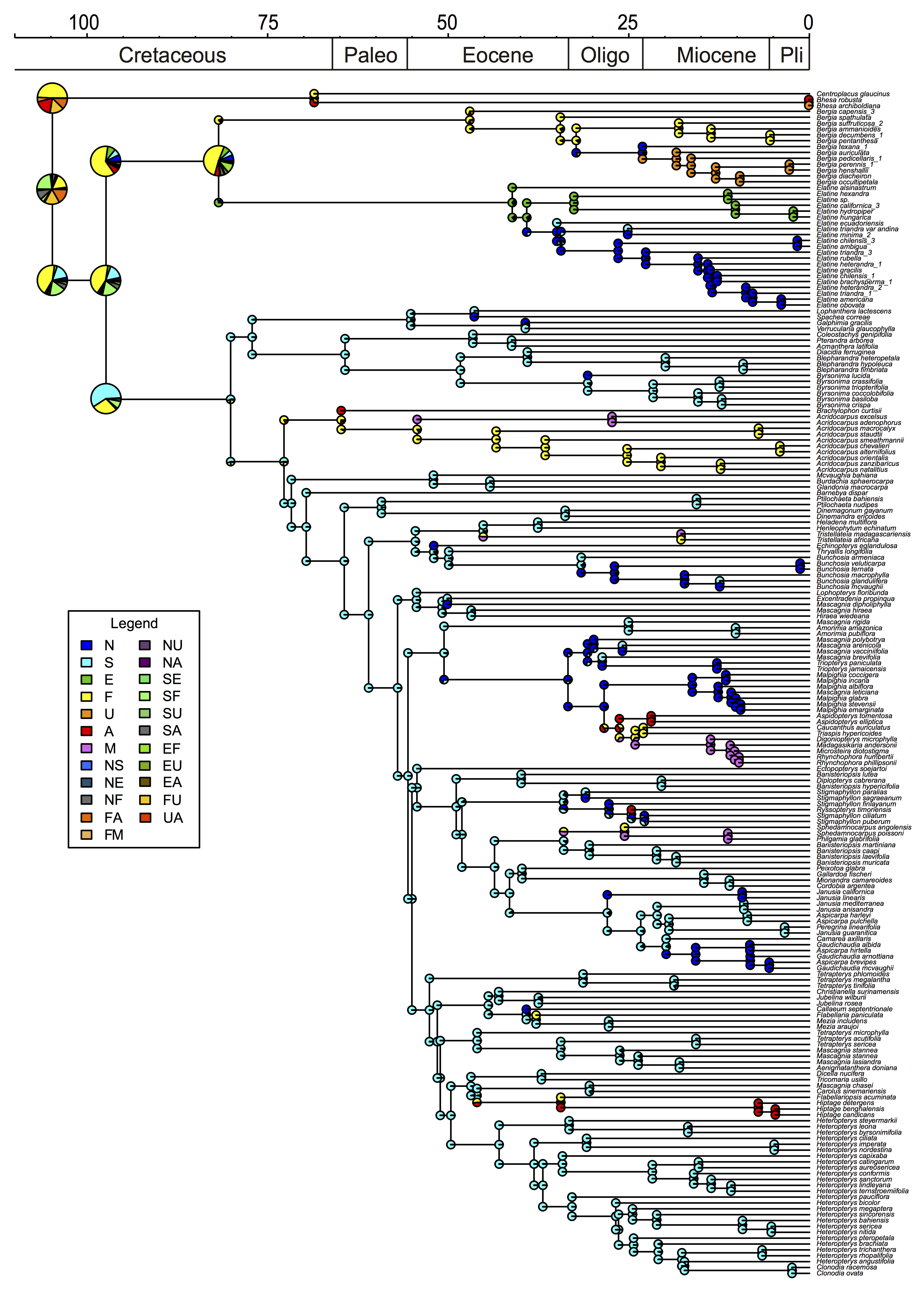

Supplement: S4 Fig — Results estimated from 100 trees sampled from the posterior distribution are summarized onto the optimum tree from the ML analysis. Pie charts at nodes show marginal log-likelihood of each geographical range scenarios. A maximum of two areas is allowed for each ancestral species. Areas are represented as follows: N = North and Central America, S = South America, E = Eurasia (Europe and Mainland Asia), F = Africa, U = Australia, Papua New Guinea, and the Pacific Islands, A = Insular Southeast Asia, including but not restricted to Malaysia, Indonesia, and the Philippines, M = Madagascar. (TIFF) [file pone.0161881.s004.tiff]

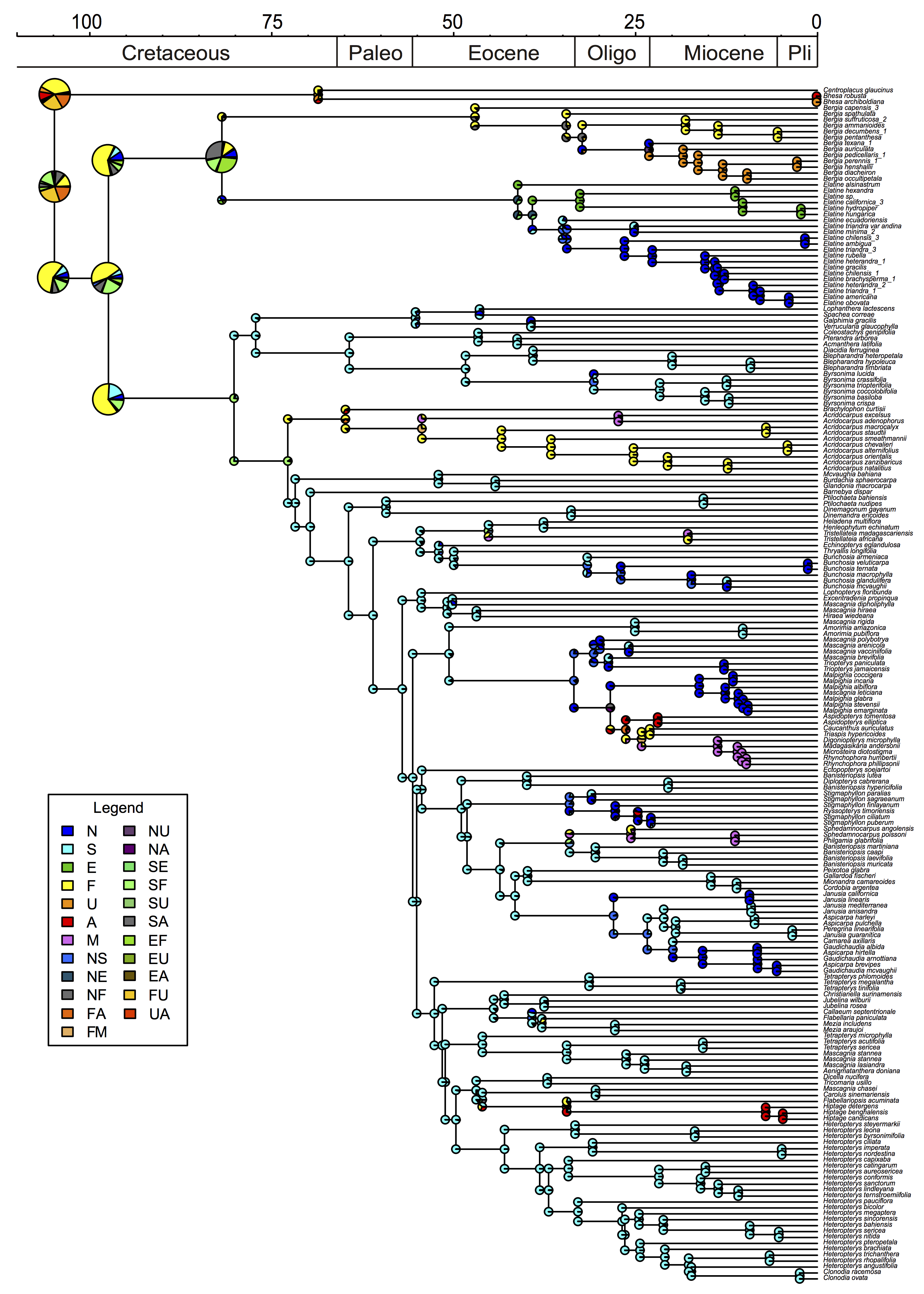

Supplement: S5 Fig — Results estimated from 100 bootstrap trees are summarized onto the optimum tree from the ML analysis. Pie charts at nodes show marginal log-likelihood of each geographical range scenarios. A maximum of two areas is allowed for each ancestral species. Areas are represented as follows: N = North and Central America, S = South America, E = Eurasia (Europe and Mainland Asia), F = Africa, U = Australia, Papua New Guinea, and the Pacific Islands, A = Insular Southeast Asia, including but not restricted to Malaysia, Indonesia, and the Philippines, M = Madagascar. (TIFF) [file pone.0161881.s005.tiff]

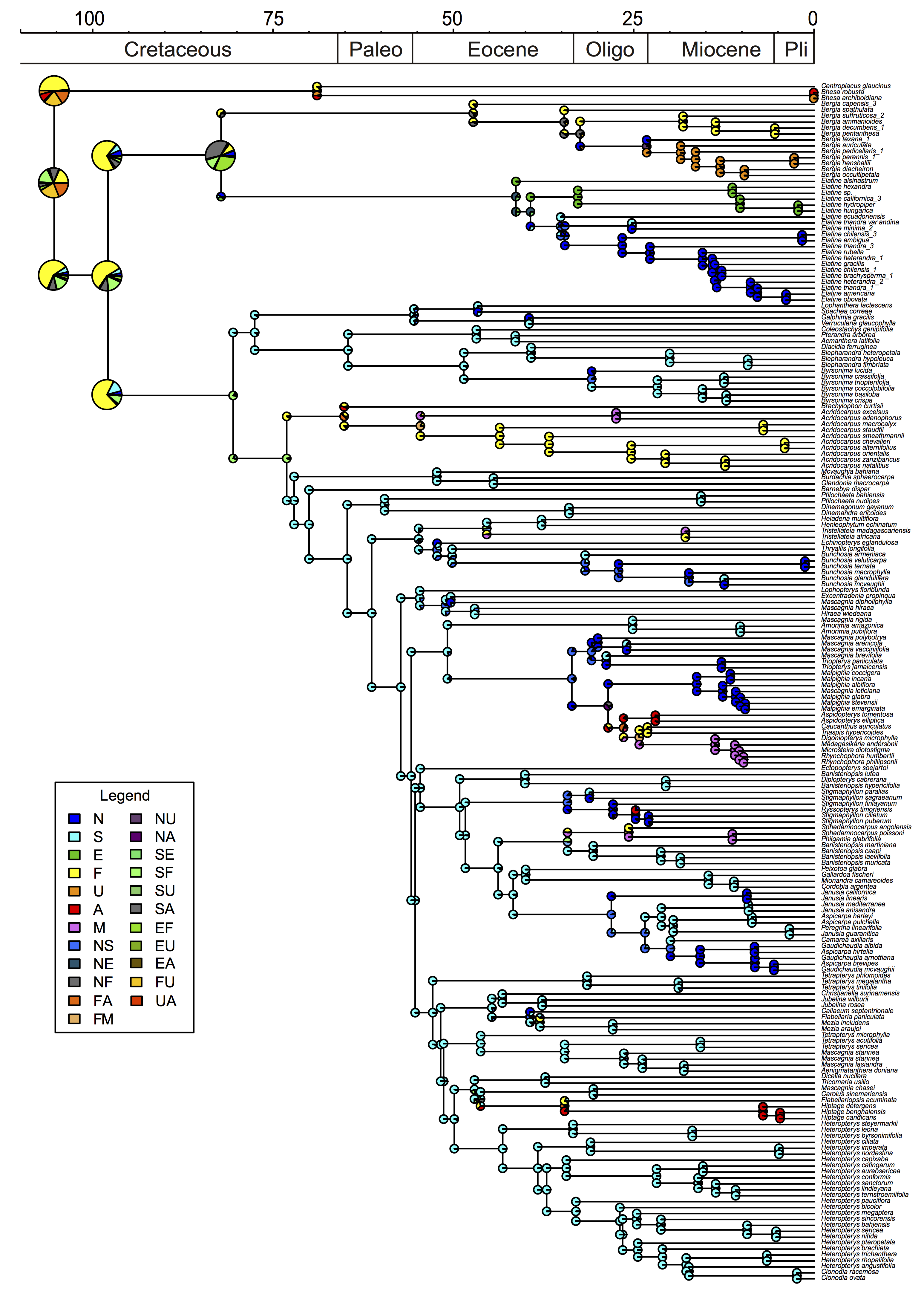

Supplement: S6 Fig — Results estimated from 100 trees sampled from the posterior distribution are summarized onto the optimum tree from ML analysis. Pie charts at nodes show marginal log-likelihood of each geographical range scenarios. A maximum of two areas is allowed for each ancestral species. Areas are represented as follows: N = North and Central America, S = South America, E = Eurasia (Europe and Mainland Asia), F = Africa, U = Australia, Papua New Guinea, and the Pacific Islands, A = Insular Southeast Asia, including but not restricted to Malaysia, Indonesia, and the Philippines, M = Madagascar. (TIFF) [file pone.0161881.s006.tiff]
