# Supplementary material for: Phylogeny of Elatinaceae and the Tropical Gondwanan Origin of the Centroplacaceae(Malpighiaceae, Elatinaceae) Clade
Source: PLoS One. 2016 Sep 29;11(9):e0161881. doi: 10.1371/journal.pone.0161881 (PMC5042423; doi:10.1371/journal.pone.0161881)
Supplement: S1 Table — “Distr.” corresponds to ranges of species applied to biogeographic analyses: N = North and Central America, S = South America, E = Eurasia (Europe and Mainland Asia), F = Africa, U = Australia, Papua New Guinea, and the Pacific Islands. “-” = data unavailable. (DOCX) [file pone.0161881.s007.docx]

**S1 Table** Taxa sequenced with distribution, voucher information, and GenBank accession numbers. “Distr.” corresponds to ranges of species applied to biogeographic analyses: N = North and Central America, S = South America, E = Eurasia (Europe and Mainland Asia), F = Africa, U = Australia, Papua New Guinea, and the Pacific Islands. “-” = data unavailable.

| **Taxon** | **Collection Locality** | **Distr.** | **Voucher** | ***ndh*F** | ***mat*K** | ***psb*A** | ***ITS*** |
| --- | --- | --- | --- | --- | --- | --- | --- |
| *Bergia ammannioides* B.Heyne ex Roth _1 | Burundi | F | M. Reekmans 9478 (MO) | KR131899 | KR131948 | KR131865 | KR131973 |
| *Bergia ammannioides* B.Heyne ex Roth _2 | Karachi | E | A. Hussain (NY) | KR131900 | - | - | - |
| *Bergia auriculata* G.J.Leach | WA, Muggon Station, Australia | U | S. Patrick 3160 (CANB) | KR131901 | KR131949 | KR131866 | KR131974 |
| *Bergia capensis* L._1 | Costa Rica | N | G. Crow 6068a (MO) | KR131902 | KR131950 | - | KR131975 |
| *Bergia capensis* L._2 | Costa Rica | N | G. Crow 6285 (MO) | KR131903 | - | - | KR131976 |
| *Bergia capensis* L._3 | Mexico | N | R. Novelo 02174 (MO) | KR131904 | KR131951 | KR131867 | KR131977 |
| *Bergia decumbens* Planch. ex Harv._1 | South Africa | F | H. Venter & J. Venter 9877 (NY) | KR131905 | KR131952 | KR131868 | KR131978 |
| *Bergia decumbens* Planch. ex Harv._2 | South Africa | F | H. Venter & J. Venter 9877 (NY) | KR131906 | - | KR131870 | KR131979 |
| *Bergia decumbens* Planch. ex Harv._3 | South Africa | F | K. Balkwill & M. Balkwill 4263 (MO) | - | KR131953 | KR131869 | KR131980 |
| *Bergia diacheiron*Verdon ex G.J.Leach | NT, Alice Springs, Australia | U | P. Latz 14791 (CANB) | KR131907 | KR131954 | KR131871 | KR131981 |
| *Bergia henshallii* G.J.Leach | NT, Darwin, Australia | U | P. Latz 13701 (CANB) | KR131908 | KR131955 | KR131872 | KR131982 |
| *Bergia occultipetala*G.J.Leach | NT, Alice Sprincgs, Australia | U | P. Latz 12446 (CANB) | KR131909 | KR131956 | KR131873 | KR131983 |
| *Bergia pedicellaris*(F.Muell.) Benth._1 | NA, Oobagooma Hsd, Australia | U | A. Mitchell 3105 (CANB) | KR131910 | KR131957 | KR131874 | KR131984 |
| *Bergia sp.* | Without specific locality | F | N. Kroon 11526 | KR131911 | KR131958 | KR131875 | KR131987 |
| *Bergia perennis*(F.Muell.) Benth._1 | NT, Australia | U | P. Fryxell, L. Craven, J. Stewart 4489 (NY) | KR131914 | - | KR131877 | KR131985 |
| *Bergia perennis*(F.Muell.) Benth._2 | NT, Australia | U | P. Fryxell, L. Craven, J. Stewart 4489 (NY) | KR131912 | - | KR131876 | KR131986 |
| *Bergia perennis*(F.Muell.) Benth._3 | NT, Australia | U | N. Ollerenshaw 590 (CANB) | KR131913 | KR131959 | - | KR131988 |
| *Bergia spathulata*Schinz | Namibia | F | O. Volk 01357 (NY) | KR131915 | KR131960 | KR131878 | KR131990 |
| *Bergia suffruticosa*Fenzl _1 | Burkina Faso | F | J. Madsen 5701 (NY) | KR131917 | - | KR131880 | KR131989 |
| *Bergia suffruticosa*Fenzl _2 | Burkina Faso | F | J. Madsen 5701 (NY) | KR131916 | - | KR131879 | - |
| *Bergia texana* Seub. ex Walp._1 | Lincoln Co., Arkansas, USA | N | R. Thomas 167,426 (NY) | KR131918 | KR131961 | KR131881 | KR131991 |
| *Bergia texana* Seub. ex Walp._2 | Lander Co., Nevada, USA | N | A. Tiehm 13075 (NY) | KR131919 | KR131962 | KR131882 | KR131992 |
| *Elatine alsinastrum* L. | Finland | E | U. Laine, T. Lammes, J. Nurmi (NY) | KR131920 | - | KR131883 | KR131993 |
| *Elatine ambigua* Wight | Without specific locality | NEF | Ahart 9476 (EIU) | KR131922 | KR131963 | KR131884 | KR131994 |
| *Elatine americana* (Pursh) Arn. | Lake Co., Montana, USA | N | C. Hitchcock, R. Spellenberg, D. Sutherland, P. Kern 24013 (NY) | KR131923 | - | KR131885 | - |
| *Elatine brachysperma* A.Gray_1 | Reno, Nevada, USA | N | M. Williams & A. Tiehm (NY) | KR131925 | - | - | KR131995 |
| *Elatine brachysperma* A.Gray_2 | Washoe, Nevada, USA | N | A. Tiehm & M. Williams 2909 (NY) | KR131926 | - | - | - |
| *Elatine brachysperma* A.Gray_3 | Reno, Nevada, USA | N | M. Williams & A. Tiehm (NY) | KR131924 | - | KR131886 | - |
| *Elatine brachysperma* A.Gray_4 | Without specific locality | N | Ahart 9524 (EIU) | KR131927 | KR131964 | KR131887 | KR131996 |
| *Elatine californica* A.Gray _1 | Riverside Co., California, USA | E | R. Thorne & E. Lathrop 37921 (NY) | KR131929 | - | - | - |
| *Elatine californica* A.Gray _2 | Lake Co., Montana, USA | E | P. Lesica 4359 (NY) | KR131930 | - | - | - |
| *Elatine californica* A.Gray _3 | Montana, USA | E | P. Lesica 4359 (NY) | KR131928 | - | KR131888 | KR131997 |
| *Elatine chilensis*Gay _1 | Elko Co., Nevada, USA | E | A. Tiehm 13061 (NY) | KR131931 | KR131965 | KR131889 | KR131998 |
| *Elatine chilensis*Gay _2 | Nevada, USA | E | A.Tiehm 13061 (NY) | KR131932 | KR131966 | KR131890 | KR131999 |
| *Elatine chilensis*Gay _3 | Without specific locality | E | Ahart 9522 (EIU) | KR131933 | KR131967 | KR131891 | KR132000 |
| *Elatine ecuadoriensis*Molau | Azuay, Ecuador | S | B. Eriksen 45747 (NY) | KR131934 | - | - | - |
| *Elatine gracilis*Mason | California, USA | N | B. Ertter, J.Strachan, L. Ahart 3292 (NY) | KR131935 | - | KR131892 | KR132001 |
| *Elatine heterandra* Mason _1 | Lake Co., California, USA | N | R. Thorne, L. DeBuhr 46826 (NY) | KR131936 | - | KR131893 | - |
| *Elatine heterandra* Mason _2 | Without specific locality | N | Ahart 4596 (MO) | KR131937 | - | - | KR132002 |
| *Elatine hexandra*DC. | Osterreich, Austria | E | T. Barta 2003-363 (NY) | KR131938 | KR131968 | KR131894 | KR132003 |
| *Elatine hungarica* Moeszi | Kazakhstan | E | A. Skvortsov, V. Bochkin, G. Klinkova, V. Sagalaev, I. Schanzer (NY) | KR131939 | - | - | KR132004 |
| *Elatine hydropiper*L._1 | South Hame, Finland | E | P. Uotila 31491 (NY) | KR131941 | - | - | - |
| *Elatine hydropiper*L._2 | Austria | E | T. Barta 2003-362 (NY) | KR131940 | KR131969 | KR131895 | KR132005 |
| *Elatine minima*(Nutt.) Fisch. & C.A.Mey._1 | Without specific locality | N | Without collector | KR131942 | KR131970 | - | KR132006 |
| *Elatine obovata* (Fassett) H.Mason | Without specific locality | N | M. Deton 1704 (MICH) | KR131943 | - | - | KR132007 |
| *Elatine rubella* Rydb. | Lander Co., Nevada, USA | N | A. Tiehm 14090 (NY) | KR131944 | KR131971 | KR131896 | KR132008 |
| *Elatine triandra* Schkuhr_1 | Salt Lake Co., Utah, USA | N | L. Arnow 5864 (NY) | KR131946 | KR131972 | - | KR132010 |
| *Elatine triandra* Schkuhr_2 | Without specific locality | - | Haynes 9547 | KR131947 | - | KR131898 | KR132011 |
| *Elatine triandra* Schkuhr var. andina Fassett | Bolivia | S | Ritter 1928 | KR131945 | - | KR131897 | KR132009 |

* Molecular data of *Elatine minima*(Nutt.) Fisch. & C.A.Mey._2, *Elatine sp.*, *Elatine triandra* Schkuhr_3, *Bergia pedicellaris* (F.Muell.) Benth._2, *Bergia texana* Seub. ex Walp._3, Malpighiaceae and Centroplacaceae was obtained from Davis *et al.* [19] and Anderson *et al*. [54].
