# Supplementary material for: Phylogeny of Elatinaceae and the Tropical Gondwanan Origin of the Centroplacaceae(Malpighiaceae, Elatinaceae) Clade
Source: PLoS One. 2016 Sep 29;11(9):e0161881. doi: 10.1371/journal.pone.0161881 (PMC5042423; doi:10.1371/journal.pone.0161881)
Supplement: S2 Table — Categories of geographical distributions were abbreviated as follows: 1) N—North and Central America; 2) S—South America; 3) E—Eurasia; 4) F—Africa; 5) U—Australia, Papua New Guinea, and the Pacific Islands; 6) A—Insular Southeast Asia, including but not restricted to Malaysia, Indonesia, and the Philippines; 7) M–Madagascar. (DOCX) [file pone.0161881.s008.docx]

**S2 Table** Time-stratified dispersal rate matrices used in the BioGeoBEARS analysis derived from [57]. Categories of geographical distributions were abbreviated as follows: 1) N—North and Central America; 2) S—South America; 3) E—Eurasia; 4) F—Africa; 5) U—Australia, Papua New Guinea, and the Pacific Islands; 6) A—Insular Southeast Asia, including but not restricted to Malaysia, Indonesia, and the Philippines; 7) M–Madagascar.

| Geological periods: 105–70 Ma | | | | | | | |
| --- | --- | --- | --- | --- | --- | --- | --- |
|  | N | S | E | F | U | A | M |
| N | 1 | 1 | 0.1 | 0.1 | 0.1 | 0.1 | 0.1 |
| S | 1 | 1 | 0.1 | 0.1 | 0.1 | 0.1 | 0.1 |
| E | 0.1 | 0.1 | 1 | 0.25 | 0.1 | 1 | 0.1 |
| F | 0.1 | 0.1 | 0.25 | 1 | 0.1 | 0.1 | 0.5 |
| U | 0.1 | 0.1 | 0.1 | 0.1 | 1 | 0.75 | 0.1 |
| A | 0.1 | 0.1 | 1 | 0.1 | 0.75 | 1 | 0.1 |
| M | 0.1 | 0.1 | 0.1 | 0.5 | 0.1 | 0.1 | 1 |

| Geological periods: 70–45 Ma | | | | | | | |
| --- | --- | --- | --- | --- | --- | --- | --- |
|  | N | S | E | F | U | A | M |
| N | 1 | 0.75 | 0.1 | 0.1 | 0.1 | 0.1 | 0.1 |
| S | 0.75 | 1 | 0.1 | 0.1 | 0.1 | 0.1 | 0.1 |
| E | 0.1 | 0.1 | 1 | 0.5 | 0.1 | 1 | 0.1 |
| F | 0.1 | 0.1 | 0.5 | 1 | 0.1 | 0.1 | 0.5 |
| U | 0.1 | 0.1 | 0.1 | 0.1 | 1 | 0.75 | 0.1 |
| A | 0.1 | 0.1 | 1 | 0.1 | 0.75 | 1 | 0.1 |
| M | 0.1 | 0.1 | 0.1 | 0.5 | 0.1 | 0.1 | 1 |

| Geological periods: 45–30 Ma | | | | | | | |
| --- | --- | --- | --- | --- | --- | --- | --- |
|  | N | S | E | F | U | A | M |
| N | 1 | 0.75 | 0.75 | 0.5 | 0.1 | 0.5 | 0.1 |
| S | 0.75 | 1 | 0.1 | 0.1 | 0.1 | 0.1 | 0.1 |
| E | 0.75 | 0.1 | 1 | 0.75 | 0.1 | 1 | 0.1 |
| F | 0.5 | 0.1 | 0.75 | 1 | 0.1 | 0.75 | 0.5 |
| U | 0.1 | 0.1 | 0.1 | 0.1 | 1 | 0.5 | 0.1 |
| A | 0.5 | 0.1 | 1 | 0.75 | 0.5 | 1 | 0.1 |
| M | 0.1 | 0.1 | 0.1 | 0.5 | 0.1 | 0.1 | 1 |

| Geological periods: 30–5 Ma | | | | | | | |
| --- | --- | --- | --- | --- | --- | --- | --- |
|  | N | S | E | F | U | A | M |
| N | 1 | 0.25 | 0.75 | 0.5 | 0.1 | 0.5 | 0.1 |
| S | 0.25 | 1 | 0.1 | 0.1 | 0.5 | 0.1 | 0.1 |
| E | 0.75 | 0.1 | 1 | 0.75 | 0.1 | 1 | 0.1 |
| F | 0.5 | 0.1 | 0.75 | 1 | 0.1 | 0.75 | 0.5 |
| U | 0.1 | 0.5 | 0.1 | 0.1 | 1 | 0.1 | 0.1 |
| A | 0.5 | 0.1 | 1 | 0.75 | 0.1 | 1 | 0.1 |
| M | 0.1 | 0.1 | 0.1 | 0.5 | 0.1 | 0.1 | 1 |

| Geological periods: 5–0 Ma | | | | | | | |
| --- | --- | --- | --- | --- | --- | --- | --- |
|  | N | S | E | F | U | A | M |
| N | 1 | 0.5 | 0.5 | 0.25 | 0.1 | 0.25 | 0.1 |
| S | 0.5 | 1 | 0.25 | 0.5 | 0.5 | 0.1 | 0.1 |
| E | 0.5 | 0.25 | 1 | 0.5 | 0.1 | 0.5 | 0.1 |
| F | 0.25 | 0.5 | 0.5 | 1 | 0.25 | 0.25 | 0.5 |
| U | 0.1 | 0.5 | 0.1 | 0.25 | 1 | 0.1 | 0.1 |
| A | 0.25 | 0.1 | 0.5 | 0.25 | 0.1 | 1 | 0.1 |
| M | 0.1 | 0.1 | 0.1 | 0.5 | 0.1 | 0.1 | 1 |
