# Supplementary material for: Phylogeny of Elatinaceae and the Tropical Gondwanan Origin of the Centroplacaceae(Malpighiaceae, Elatinaceae) Clade
Source: PLoS One. 2016 Sep 29;11(9):e0161881. doi: 10.1371/journal.pone.0161881 (PMC5042423; doi:10.1371/journal.pone.0161881)
Supplement: S3 Table — (DOCX) [file pone.0161881.s009.docx]

| **Node** | **Age estimations from treePL with 95% CI (Ma)** | **Age estimations from BEAST with 95%HPD (Ma)** |
| --- | --- | --- |
| Root | 105 | 108(106.1,110.1) |
| Stem group Malp+Elat | 98(94.5,99.2) | 107(103.9,110.0) |
| Crown group Elat | 82(74.0,87.1) | 85(73.6,96.8) |
| Crown group Malp | 80(70.6,82.2) | 88(82.4,93.2) |
| Crown group Bergia | 47(35.7,56.6) | 54(40.0,70.3) |
| Crown group Elatine | 41(20.9,45.6) | 37(27.0,47.3) |

**S3 Table** Age estimations for early divergence events within (Centroplacaceae(Malpighiaceae,Elatinaceae)) clade using treePL and BEAST.
