# Supplementary material for: Phylogeny of Elatinaceae and the Tropical Gondwanan Origin of the Centroplacaceae(Malpighiaceae, Elatinaceae) Clade
Source: PLoS One. 2016 Sep 29;11(9):e0161881. doi: 10.1371/journal.pone.0161881 (PMC5042423; doi:10.1371/journal.pone.0161881)
Supplement: S4 Table — (DOCX) [file pone.0161881.s010.docx]

**S4 Table** Estimated likelihood for biogeographic reconstruction with input phylogenies from treePL/BEAST under DEC/DEC+J model

| **Dating Methods** | **Range Evolution Model** | **lnL** | **Dispersal parameter (d)** | **Extinction parameter (e)** | **j** |
| --- | --- | --- | --- | --- | --- |
| treePL | DEC+J | -218.27 | 0.00196 | 1e-12 | 0.0794 |
| treePL | DEC | -262.70 | 0.00196 | 1e-12 | 0 |
| BEAST | DEC+J | -217.42 | 0.00196 | 1e-12 | 0.0794 |
| BEAST | DEC | -255.57 | 0.00196 | 1e-12 | 0 |
