# Supplementary material for: Phylogeny of Elatinaceae and the Tropical Gondwanan Origin of the Centroplacaceae(Malpighiaceae, Elatinaceae) Clade
Source: PLoS One. 2016 Sep 29;11(9):e0161881. doi: 10.1371/journal.pone.0161881 (PMC5042423; doi:10.1371/journal.pone.0161881)
Supplement: S5 Table — (DOCX) [file pone.0161881.s011.docx]

**S5 Table** Comparison of the phylogenetic results from this study with the classification of Elatinaceae sensu Niedenzu (1925); * denotes a monotypic taxon.

| **Genus** | **Subgenus** | **Section** | **Phylogenetic Status** |
| --- | --- | --- | --- |
| *Bergia* L. |  |  | Monophyletic |
|  | n.a. |  | n.a. |
|  |  | *Dichasianthae* Niedz | Non-monophyletic |
|  |  | *Monanthae* Niedz. (Groups 1–4 *sensu* Leach) | Monophyletic |
| *Elatine* L. |  |  | Monophyletic |
|  | *Hydropiper* Moesz. |  | Monophyletic |
|  |  | *Crypta* Seub. | Non-monophyletic |
|  |  | *Elatinella* Seub. | Non-monophyletic |
|  | *Potamopitys* Seub. |  | Monophyletic* |
|  |  | n.a. | n.a. |
